# Antioxidant constituents of three selected red and green color *Amaranthus* leafy vegetable

**DOI:** 10.1038/s41598-019-52033-8

**Published:** 2019-12-03

**Authors:** Umakanta Sarker, Shinya Oba

**Affiliations:** 1 Department of Genetics and Plant Breeding, Faculty of Agriculture, Bangabandhu Sheikh Mujibur Rahman Agricultural University, Gazipur, 1706 Bangladesh; 2 Laboratory of Field Science, Faculty of Applied Biological Sciences, Gifu University, Yanagido 1-1, Gifu, Japan

**Keywords:** Biochemistry, Natural variation in plants

## Abstract

Red color (*A*. *tricolor*) genotypes are an excellent source of pigments, such as betalain (1122.47 ng g^−1^ FW), β-xanthin (585.22 ng g^−1^ FW), β-cyanin (624.75 ng g^−1^ FW), carotenoids (55.55 mg 100 g^−1^ FW), and antioxidant phytochemicals, such as vitamin C (122.43 mg 100 g^−1^ FW), TFC (312.64 RE µg g^−1^ DW), TPC (220.04 GAE µg g^−1^ DW), TAC (DPPH and ABTS^+^) (43.81 and 66.59 TEAC µg g^−1^ DW) compared to green color (*A*. *lividus*) genotype. Remarkable phenolic acids, such as salicylic acid, vanillic acid, protocatechuic acid, gallic acid, gentisic acid, β-resorcylic acid, *p*-hydroxybenzoic acid, syringic acid, ellagic acid, chlorogenic acid, sinapic acids, *trans*-cinnamic acid, *m*-coumaric acid, caffeic acid, *p*-coumaric acid, ferulic acid, and flavonoids, such as rutin, hyperoside, isoquercetin, myricetin, quercetin, apigenin, kaempferol, and catechin were observed in the red color amaranth genotypes, which was much higher compared to the green color amaranth genotype. We newly identified four flavonoids such as quercetin, catechin, myricetin, and apigenin in amaranth. Among the three selected advanced genotypes studied the red color genotype VA13 and VA3 had abundant antioxidant pigments, phytochemicals, phenolic acids, flavonoids, and antioxidant activity could be selected for extracting colorful juice. Correlation study revealed that all antioxidant constituents of red color amaranth had strong antioxidant activity. The present investigation revealed that two red color genotypes had an excellent source of antioxidants that demand detail pharmacological study.

## Introduction

*Amaranthus* belongs to the family *Amaranthaceae* and consists of 70 species. Among them, 17 species are used as edible leaves and 3 are used as food grains^1^. These plants are fast growing grains, vegetables, and ornamental plants, widely distributed in America, Africa, Australia, Asia, and Europe. *Amaranthus* leaves and stems are inexpensive and excellent sources of dietary fiber, protein with essential amino acids, such as lysine and methionine, carotenoids, vitamin C, and minerals, such as calcium, magnesium, potassium, phosphorus, iron, zinc, copper, and manganese^2–8^. Members of these genera are widely used as traditional medicinal plants, especially as antiviral, antimalarial, antidiabetic, antibacterial, antihelminthic and snake antidote^9–11^. *Amaranthus* leaves contain a unique source of antioxidant pigments, such as betalain, β-xanthin, and β-cyanin compared to other leafy vegetables and an excellent source of other antioxidant pigments, such as anthocyanins, carotenoids, and chlorophylls^12,13^, and natural antioxidant phytochemicals, such as vitamin C, phenolic acids, and flavonoids^14^. These natural antioxidant compounds are not only significant for the food industry because of their health-promoting effects, but also as natural preservatives of food products^15–17^. Recently, natural antioxidants of vegetables attracted consumers and researchers. These natural antioxidants defense against several diseases, such as cardiovascular diseases, cancer, cataracts, atherosclerosis, retinopathy, arthritis, emphysema, and neuro-degenerative diseases^18–20^. It is tolerant to abiotic stresses, such as drought^21–24^ and salinity^25–27^.

There are two colors in amaranth, one is red and another is green^28^. Red amaranth has more pigments than green amaranth. Bangladesh has a lot of amaranth germplasms with great variability and phenotypic plasticity^29^ that has multipurpose uses. *A*. *tricolor and A*. *lividus* are a cheap and popular leafy vegetable in Bangladesh including south-east Asia, Africa, and South America. Its nutritional value, taste, and attractive leaf color make them very popular in the Asian continent and the globe. Amaranth is grown year-round and even though in the gaps of foliage crops between winter and hot summer^3,4^.

Recently, we were evaluating the chances of utilizing antioxidant pigments of amaranth along with the phenolic profile of interest for making drinks^12,13^. In our earlier studies, we screened 24 *A*. *tricolor* genotypes based on yield, yield contributing traits as well as total antioxidant capacity and selected the best yielding and antioxidant potential *A*. *tricolor* genotype VA3 and VA13 as advanced genotypes (Data not published). In another study, we screened 96 *A*. *lividus* genotypes based on yield and yield contributing traits and selected the best yielding *A*. *lividus* genotype GRA1 as advanced genotype (Data not published). Hence, in this study, we evaluate the pigments, phytochemicals, phenolic profile, and the antioxidant of three advanced leafy vegetable genotypes in detail through spectrophotometry, HPLC, and LC-MS to select appropriate genotypes for extracting the juice.

## Results and Discussion

### Parameters of leaf color

**Figure 1:**
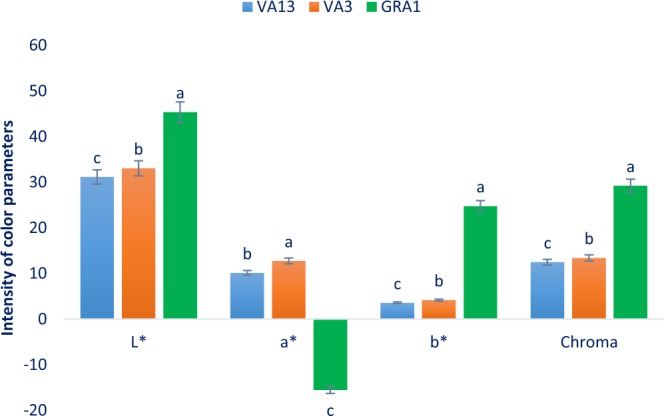
Leaf color parameters in three selected red and green color *Amaranthus* leafy vegetable, L*, Lightness; a*, Redness/greenness; b*, Yellowness/blueness, different letters in the bar are differed significantly by Duncan Multiple Range Test (P < 0.001), (n = 3).

Leaf color parameters of three selected red and green color *Amaranthus* leafy vegetables are presented in Fig. 1. The significant variations were observed for chroma, a*, lightness (L*), and b* of the three genotypes studied. Chroma, a*, lightness (L*), and b* ranged from 12.46 to 29.22, −15–52 to 12.75, 31.16 to 45.36, and 3.56 to 24.76, respectively. The green color genotype GRA1 had the highest lightness value, while the lowest lightness was recorded in the red color genotype VA13 followed by VA3. Similarly, the highest b* and chroma value was noticed in the genotype GRA1 and the lowest b* and chroma value was recorded in the genotype VA13 followed by VA3. The highest a* value (12.75) was observed in the genotype VA3, while GRA1 showed the lowest a* value. Our results were in full agreement with the results obtained from Colonna *et al*.^30^. Leaf color is an essential parameter that has a significant contribution to acceptability, preference, and consumer’s choice. It is considered as a key indicator of the leafy vegetables for evaluating the antioxidant potentiality^30^. Red color genotype VA13 and VA3 had high redness and yellowness values indicating the presence of abundant pigments (β-xanthin, anthocyanins, β-cyanin, betalain, and carotenoids). In contrast, the green color genotype showed high greenness and blueness indicating the presence of minimum pigments (β-xanthin, anthocyanins, β-cyanin, betalain, and carotenoids). The red genotype had bright red-violet color due to the presence of abundant betacyanin pigments with better stability at low temperatures (<14 °C) at pH 5–7^31^. These red color genotypes could be used for making colorful juice, as colorants and natural preservatives of food products.

### Antioxidants leaf pigments

**Figure 2:**
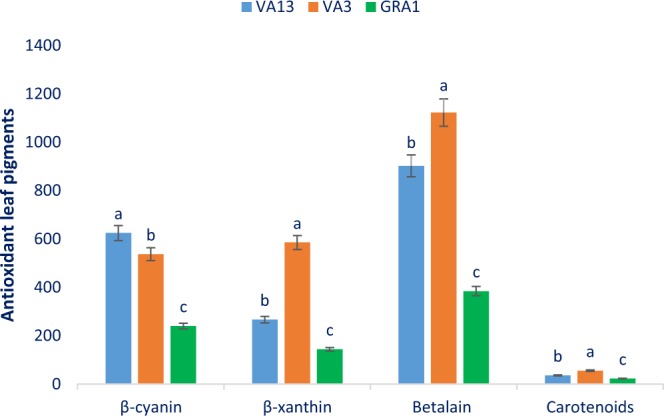
Antioxidant leaf pigments in three selected red and green color *Amaranthus* leafy vegetable, β-cyanin (ng g^−1^ FW), β-xanthin (ng g^−1^ FW), Betalain (ng g^−1^ FW), Carotenoids (mg 100 g^−1^ FW), different letters in the bar are differed significantly by Duncan Multiple Range Test (P < 0.001), (n = 3).

Antioxidant leaf pigments of three selected red and green color *Amaranthus* leafy vegetables are shown in Fig. 2. Betalain, β-xanthin, and β-cyanin varied significantly and remarkably with the genotypes and ranged from 384.39 to 1122.47, 144.11 to 585.22, and 240.28 to 624.75 ng g^−1^ FW, respectively. The red color genotype VA13 exhibited the highest β-cyanin content, followed by VA3. Among genotypes, significant and remarkable variations were observed in β-xanthin content. Betalain and β-xanthin content were the highest in the red color genotype VA3 followed by VA13. In contrast, the green color genotype GRA1 exhibited the lowest β-cyanin, β-xanthin, and betalain content. Pronounced variations were observed in the carotenoids content of the genotypes. Carotenoids content ranged from 23.02 to 55.55 mg 100 g^−1^ FW. The red color genotype VA3 exhibited the highest carotenoids, while the lowest carotenoids were recorded in the green color genotype GRA1. The red color genotype VA13 and VA3 had high carotenoids pigments compared to the green color genotype GRA1. In this study, we found remarkable carotenoids, β-cyanin, betalain, and β-xanthin in the red color genotype compared to the green color genotype, which was corroborated with the results of red and green amaranth of Khanam and Oba^32^. We obtained two to three fold greater carotenoids contents in red color genotypes compared to the carotenoids contents of *A*. *gangeticus* genotype of Raju *et al*.^33^. The leaf carotenoids contents of red color genotypes two to three fold and green color genotype were 50% greater than the carotenoids contents of the leaves of *A*. *caudatus*^15^. The betalain content of our study was also corroborated with the betalain content of Li *et al*.^34^ where they reported the highest betalain content in *A*. *caudatus* leaves compared to *A*. *hypochondriacus* leaves. They also reported that leaves had the highest betalain compared to different parts of plants (seed, stalks, sprouts, flowers). Generally, the red genotype with darker or deeper red-violet color showed higher pigments (betalain, carotenoids, β-cyanin, β-xanthin) as well as greater antioxidant activity^31^. In the present study, color parameters of three red and green color *Amaranthus* leafy vegetables were also related to antioxidant pigments and phytochemical contents. Specifically, relative lower lightness (L*) values, higher redness value (a*), were found in red color *Amaranthus* leafy vegetable that had higher pigments (betalain, carotenoids, β-cyanin, β-xanthin). The genotype VA13 and VA3 had abundant antioxidant pigments with free radical-scavenging activity^17^. Hence, the red color genotype VA13 and VA3 could be consumed in our daily diet for human health benefits as these pigments played an essential role in detoxification of ROS in the human body and preventing many degenerative human diseases and antiaging^15,16^.

### Antioxidant phytochemicals

**Figure 3:**
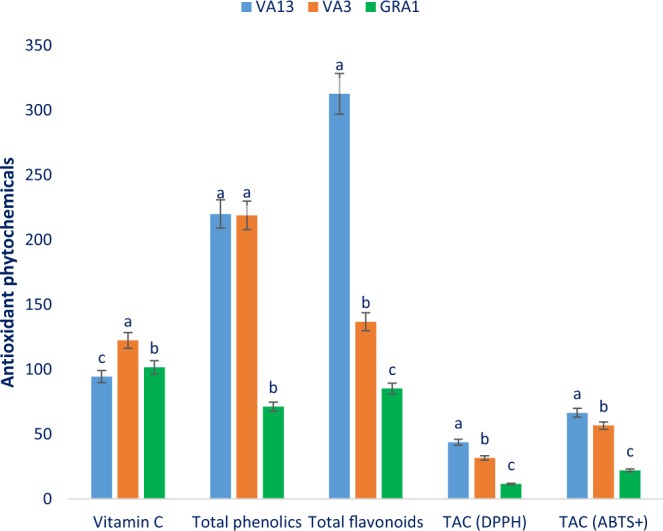
Vitamin C, total phenolics, total flavonoids, and free radical scavenging capacity in three selected red and green color *Amaranthus* leafy vegetable, vitamin C (mg 100 g^−1^ FW), TPC, Total polyphenol content (GAE µg g^−1^ DW); TFC, Total flavonoid content (RE µg g^−1^ DW); TAC (DPPH), Total antioxidant capacity (DPPH) (TEAC µg g^−1^ DW); TAC (ABTS^+^), Total antioxidant capacity (ABTS^+^) (TEAC µg g^−1^ DW); different letters in the bar are differed significantly by Duncan Multiple Range Test (P < 0.001), (n = 3).

Total phenolic compounds (TPC), vitamin C, total flavonoid compounds (TFC) and total antioxidant activity (TAC) varied significantly among the studied genotype (Fig. 3). Vitamin C content ranged from 94.51 mg 100 g^−1^ FW in the genotype VA13 to 122.43 mg 100 g^−1^ FW in the genotype VA3. Total phenolic compounds (TPC) ranged from 71.62 GAE µg g^−1^ DW (GRA1) to 220.04 GAE µg g^−1^ DW (VA13). The red color genotype VA13 had the highest TPC which is statistically similar to VA3. TFC exhibited much noticeable variation over genotypes, which ranged from 85.27 RE µg g^−1^ DW in the genotype GRA1 to 312.65 RE µg g^−1^ DW in the genotype VA13. The red color genotype VA13 exhibited the highest TFC showing the following order: VA13 ˃ V3 ˃ GRA1. TAC (DPPH) ranged from 11.62 TEAC µg g^−1^ DW (GRA1) to 43.81 TEAC µg g^−1^ DW (VA13). The highest TAC (DPPH) was recorded in the red color genotype VA13 followed by VA3. In contrast, GRA1 had the lowest TAC (DPPH). TAC (ABTS^+^) with a range of 22.24 TEAC µg g^−1^ DW to 66.59 TEAC µg g^−1^ DW. The red color genotype VA13 had the highest TAC (ABTS^+^). In contrast, TAC (ABTS^+^) was the lowest in green color genotype GRA1. The red color genotype VA13 and VA3 contained higher vitamin C, TPC, TFC, and TAC compared to green color genotype GRA1. Our results were fully agreed to the results of Khanam and Oba^32^ where they observed higher TPC, TFC, and TAC content in the red color amaranth genotype compared to green color amaranth. Similarly, our observed vitamin C was much greater than vitamin C reported by Jiminez-Aguilar and Grusak^35^ in fifteen different species of *Amaranthus* leafy vegetables. Li *et al*.^34^ noticed the highest total polyphenol content, total flavonoids content and total antioxidant activity (FRAP and ORAC methods) in *A*. *hypochondriacus* leaves compared to *A*. *caudatus* leaves. They also reported that leaves had the highest total polyphenol content, total flavonoids content and total antioxidant activity (FRAP) compared to different parts of plants (seed, stalks, sprouts, flowers). Kraujalis *et al*.^36^ reported the highest TPC and antioxidant capacity (ABTS+, DPPH, and ORAC) in the leaves compared to other parts (Stem, flower, seed) of *Amaranthus hybridus*. It is difficult to compare with our present results due to the difference in extraction and estimation methods and standard references. Color parameters of red and green color genotypes were also related to antioxidant pigments and antioxidant activity. Red *Amaranthus* leafy vegetables with darker or deeper red-violet color having higher antioxidants pigments and phytochemicals such as vitamin C, TPC, and TFC showed greater antioxidant activity^31^. In the present study, antioxidant phytochemicals such as TPC and TFC were measured by spectrophotometric methods to compare with HPLC detected total polyphenols, flavonoids and total phenolic index (TPI). In this study, an accurate analysis of individual compounds was done through HPLC. Therefore, the total phenolic index (TPI) has been proposed as an alternative and complementary approach to the TPC^34^.

In this study, we found remarkable pigments such as carotenoids, β-cyanin, betalain, β-xanthin, and antioxidant phytochemicals such as TFC, vitamin C, TPC, and antioxidant potential in the red color amaranth genotypes, which was much higher compared to green color amaranth genotype. Our results were fully agreed to the results of Khanam and oba^32^ where they observed higher TAC, carotenoids, β-cyanin, TFC, betalain, β-xanthin, and TPC content in the red amaranth genotype compared to green amaranth. Pigments such as betalain (1122.47 ng g^−1^ FW), β-cyanin (624.75 ng g^−1^ FW), carotenoids (55.55 mg 100 g^−1^ FW), β-xanthin (585.22 ng g^−1^ FW), and antioxidant phytochemicals such as TAC (ABTS^+^) (66.59 TEAC µg g^−1^ DW), TFC (312.64 RE µg g^−1^ DW), and TAC (DPPH) (43.81 TEAC µg g^−1^ DW) obtained in this study were corroborated with the results of Khanam *et al*.^37^ in *A*. *tricolor*. The red color genotypes VA13 and VA3 had abundant pigments such as carotenoids, β-cyanin, betalain, β-xanthin, and antioxidant phytochemicals such as TAC, vitamin C, TFC, and TPC. The genotypes VA13 and VA3 could be used as antioxidant profile enriched high-yielding varieties. The present investigation revealed that these two genotypes have an excellent source of phenolics, flavonoids, vitamins, antioxidant leaf pigments, and antioxidants which were much greater than the results of Khanam and Oba^32^ and Khanam *et al*.^37^ that offered huge prospects for feeding the vitamin and antioxidant deficient community.

### Flavonoids and phenolic acids

**Figure 4:**
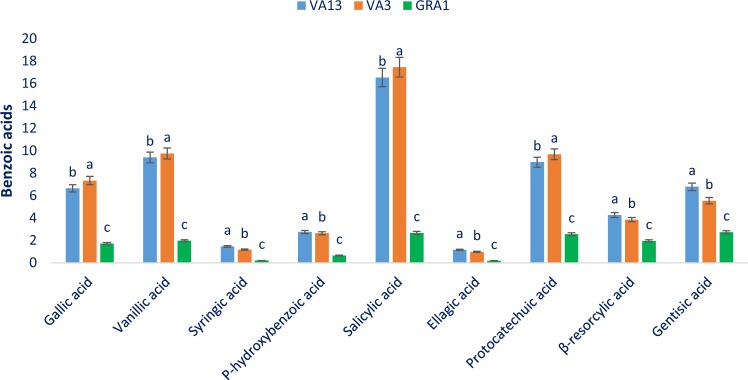
Benzoic acid compositions (µg g^−1^ FFW) in three selected red and green color *Amaranthus* leafy vegetable, different letters in the bar are differed significantly by Duncan Multiple Range Test (P < 0.001), (n = 3).

**Figure 5:**
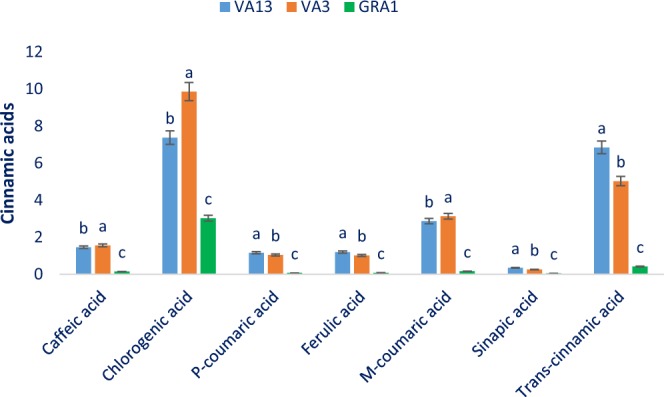
Cinnamic acid compositions (µg g^−1^ FFW) in three selected red and green color *Amaranthus* leafy vegetable, different letters in the bar are differed significantly by Duncan Multiple Range Test (P < 0.001), (n = 3).

**Figure 6:**
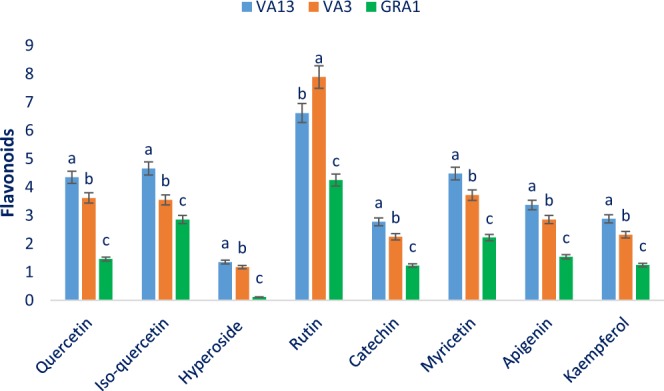
Flavonoids compositions (µg g^−1^ FFW) in three selected red and green color *Amaranthus* leafy vegetable, different letters in the bar are differed significantly by Duncan Multiple Range Test (P < 0.001), (n = 3).

**Figure 7:**
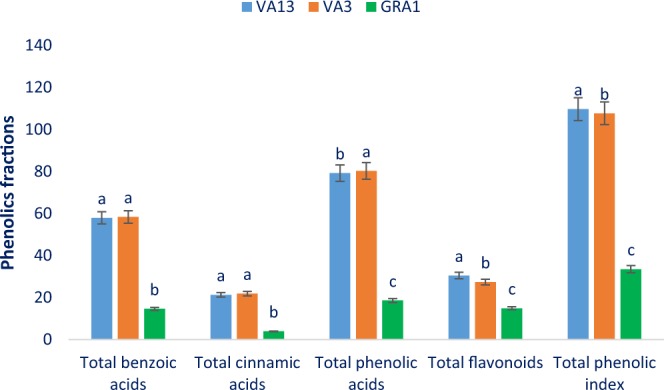
Phenolic fractions (total benzoic acids, total cinnamic acids, total phenolic acids, total flavonoids, and total phenolic index) (µg g^−1^ FFW) in three selected red and green color *Amaranthus* leafy vegetable, different letters in the bar are differed significantly by Duncan Multiple Range Test (P < 0.001), (n = 3).

Table 1 represents the data on the molecular ion, main fragment ions in MS^2^, λmax, retention time, and identified compounds. The LC separated flavonoids and phenolic acid values from three genotypes (VA13, VA3, and GRA1) were compared with standard flavonoids and phenolic acid masses through the corresponding peaks of the compounds. A total of twenty-four phenolic compounds were identified. Among them, nine benzoic acids, seven cinnamic acids, and eight flavonoids compounds. We identified four flavonoids (quercetin, catechin, myricetin, and apigenin) compounds in red and green color *Amaranthus* leaves for the first time. Except for these nine flavonoids and phenolic acids, Khanam and Oba^32^, Khanam *et al*.^37^ in red and green amaranths reported the rest 15 flavonoids and phenolic acids. Li *et al*.^34^ reported 11 phenolic compounds such as gallic acid, protocatechuic acid, chlorogenic acid, gentisic acid, β-resorcylic acid, ferulic acid, salicylic acid, ellagic acid, rutin, quercetin, and kaempferol in different parts (Leaf, seed, stalks, sprouts, flowers) of *A*. *hypochondriacus*, *A*. *cruentus*, and *A*. *caudatus*. Pasko *et al*.^38^ identified 8 phenolic acids such as gallic acid, *p*-hydroxybenzoic acid, vanillic acid, *p*-coumaric acid, syringic acids, ferulic acid, caffeic acids, cinnamic acids and 3 flavonoids such as rutin, vitexin, isovitexin in the sprouts and seeds of *A*. *cruentus* (Aztec and Rawa). Figures 4–7 represent the identified phenolic compounds of three selected red and green color *Amaranthus* leaves. Among three major groups of compounds, benzoic acids were the most abundant compounds followed by flavonoids in three studied genotypes (Figs. 4–7).

**Table 1:** Retention time (Rt), wavelengths of maximum absorption in the visible region (λ_max_), mass spectral data and tentative identification of phenolic compounds in three selected red and green *Amaranthus* leafy vegetable.

Peak no	Rt (min)	λ_max_ (nm)	Molecular ion [M - H] (m/z)	MS^2^ (m/z)	Identity of tentative compounds
1	9.1	254	169.1142	169.1563	3,4,5 Trihydroxybenzoic acid
2	30.6	254	167.1214	167.1564	4-Hydroxy-3-methoxybenzoic acid
3	34.8	254	197.1132	197.1104	3,5-Dimethoxy-4-hydroxybenzoic acid
4	31.5	254	137.0213	137.1574	4-Hydroxybenzoic acid
5	48.2	254	137.2113	137.1582	2-Hydroxybenzoic acid
6	52.5	254	301.0423	301.0643	2,3,7,8-Tetrahydroxy-chromeno [5,4,3-cde] chromene-5,10-dione
7	2.2	280	154.1212	154.1157	3,4-Dihydroxybenzoic acid
8	4.0	280	154.1212	154.0156	2,4-Dihydroxybenzoic acid
9	3.7	280	154.1212	154.1157	2,5- Dihydroxybenzoic acid
10	32.0	280	179.0821	179.0687	3,4-Dihydroxy-trans-cinnamate
11	31.1	280	353.1253	353.1542	3-(3,4-Dihydroxy cinnamoyl) quinic acid
12	42.0	280	163.0658	163.1241	4-Hydroxy cinnamic acid
13	47.9	280	193.1726	193.1649	3-Methoxy-4-hydroxy cinnamic acid
14	49.6	280	163.2547	163.2872	3-Hydroxy cinnamic acid
15	49.0	280	223.1568	223.1748	4-Hydroxy-3,5-dimethoxy cinnamic acid
16	67.3	280	147.1142	147.1103	3-Phenyl acrylic acid
17	23.9	280	290.2463	290.1238	(2R-3S)-2-(3,4-dihydroxyphenyl)-3,4-dihydro-2-chromene-3,5,7-triol
18	54.3	360	463.2875	463.3124	Quercetin-3-*O*-glucoside
19	53.3	360	463.4358	463.5125	Quercetin-3-*O*-galactoside
20	53.0	360	609.3874	609.4265	Quercetin-3-*O*-rutinoside
21	7.5	370	301.0348	301.2267	2-(3,4-dihydroxy phenyl)-3,5,7-trihydroxychromene-4-one
22	4.6	370	626.2468	626.3216	Myricetin-3-*O*-rutinoside
23	15.4	370	270.2344	270.3221	4′,5,7-Trihydroxyflavone, 5,7-Dihydroxy-2-(4-hydroxyphenyl)-4-benzopyron
24	17.8	370	593.4253	593.3687	kaempferol-3-*O*-rutinoside

Within benzoic acids, salicylic acid was noticed as the most abundant phenolic acids followed by protocatechuic acid, vanillic acid, gallic acid, gentisic acid, and β-resorcylic acid (Fig. 4). Our obtained *p*-hydroxybenzoic acid and gallic acid content in the red color genotype VA13 and VA3 were greater than the results of Khanam *et al*.^37^ in.*A*. *tricolor*. Vanillic acid, salicylic acid, protocatechuic acid, and gallic acid ranged from 1.97 to 9.75, 2.67 to 17.45, 2.55 to 9.68, and 1.72 to 7.23 µg g^−1^ FFW, respectively (Fig. 4). The highest salicylic acid (17.45 µg g^−1^ FFW), vanillic acid (9.75 µg g^−1^ FFW), protocatechuic acid (9.68 µg g^−1^ FFW), and gallic acid (7.23 µg g^−1^ FFW) were obtained from the red color genotype VA3 followed by the genotype VA13. Whereas the green color genotype GRA1 showed the lowest salicylic acid (2.67 g g^−1^ FFW), vanillic acid (1.97 µg g^−1^ FFW), protocatechuic acid (2.55 µg g^−1^ FFW), and gallic acid (1.72 µg g^−1^ FFW). Gentisic acid, β-resorcylic acid, *P*-hydroxy benzoic acid, syringic acid, and ellagic acid ranged from 2.73 to 6.68, 1.96–4.26, 0.65 to 2.75, 0.21 to 1.46, and 0.18 to 1.16 µg g^−1^ FFW, respectively (Fig. 4). The red color genotype VA13 exhibited the highest gentisic acid (6.68 µg g^−1^ FFW), β-resorcylic acid (4.26 µg g^−1^ FFW), *P*-hydroxy benzoic acid (2.75 µg g^−1^ FFW), syringic acid (1.46 µg g^−1^ FFW) and ellagic acid (1.16 µg g^−1^ FFW) followed by the red color genotype VA3, while the green color genotype GRA1 had the lowest gentisic acid (2.73 µg g^−1^ FFW), β-resorcylic acid (1.96 µg g^−1^ FFW), *P*-hydroxy benzoic acid (0.65 µg g^−1^ FFW), syringic acid (0.21 µg g^−1^ FFW) and ellagic acid (0.18 µg g^−1^ FFW).

Considering cinnamic acids, the most pronounced compound was chlorogenic acid followed by *m*-coumaric acid and *trans*-cinnamic acid (Fig. 5). Red color genotypes had abundant ferulic acid, *p*-coumaric acid, and caffeic acid. The *m*-coumaric acid and caffeic acid of red color genotype VA13 and VA3 were found to be higher compared to the reported results of Khanam *et al*.^37^ in *A*. *tricolor*. Caffeic acid, *m*-coumaric acid, and chlorogenic acid ranged from 0.15 to 1.56, 0.16 to 3.13, and 3.03 to 9.86 µg g^−1^ FFW, respectively (Fig. 5). Caffeic acid, *m*-coumaric acid, and chlorogenic acid were the highest (1.56 µg g^−1^ FFW, 3.13 µg g^−1^ FFW, and 9.86 µg g^−1^ FFW) in the red color genotype VA3 followed by the genotype VA13. However, the green color genotype GRA1 exerted the lowest caffeic acid, *m*-coumaric acid, and chlorogenic acid (0.15 µg g^−1^ FFW, 0.16 µg g^−1^ FFW, and 3.03 µg g^−1^ FFW). Ferulic acid, sinapic acid, *trans*-cinnamic acid, and *p*-coumaric acid ranged from 0.08 to 1.20, 0.05 to 0.35, 0.42 to 6.85, and 0.07 to 1.16 µg g^−1^ FFW (Fig. 5). The highest ferulic acid, sinapic acid, *trans*-cinnamic acid, and *p*-coumaric acid (1.20 µg g^−1^ FFW, 0.35 µg g^−1^ FFW, 6.85 µg g^−1^ FFW, and 1.16 µg g^−1^ FFW) were reported in the red color genotype VA13 followed by the red color genotype VA3. In contrast, the green color genotype GRA1 had the lowest *trans*-cinnamic acid, sinapic acid, ferulic acid, and *p*-coumaric (0.42 µg g^−1^ FFW, 0.05 µg g^−1^ FFW, 0.08 µg g^−1^ FFW and 0.07 µg g^−1^ FFW).

In the present study, red color genotypes VA13 and VA3 had abundant flavonoids such as rutin, isoquercetin, myricetin, quercetin, apigenin, kaempferol, and catechin. Isoquercetin, hyperoside, rutin, myricetin, quercetin, apigenin, kaempferol, and catechin ranged from 2.86 to 4.66, 0.12 to 1.35, 4.25 to 7.89, 2.22 to 4.48, 1.46 to 4.38, 1.54 to 3.37, 1.25 to 2.88, and 1.23 to 2.78 µg g^−1^ FFW, respectively (Fig. 6). The red color genotype VA13 exhibited the highest isoquercetin, myricetin, quercetin, apigenin, kaempferol, catechin, and hyperoside (4.66, 4.25, 4.48, 4.38, 3.37, 2.88, 2.78, and 1.35 µg g^−1^ FFW, respectively) followed by VA3, while the lowest isoquercetin, myricetin, quercetin, apigenin, kaempferol, catechin, and hyperoside (2.86, 2.22, 1.46, 1.54, 1.25, 1.23, and 0.12 µg g^−1^ FFW, respectively) was observed in the green color genotype GRA1. The highest rutin was reported in the red color genotype VA3 (7.89 µg g^−1^ FFW) followed by VA13, albeit the green color genotype GRA1 had the lowest rutin (4.25 µg g^−1^ FFW) (Fig. 6). Isoquercetin, hyperoside of our red color genotypes were higher than the content of *A*. *tricolor* genotypes reported by Khanam *et al*.^37^.

Total benzoic acids, total cinnamic acids, total phenolic acids, total flavonoids, and total phenolic index ranged from 14.64 to 58.40, 3.96 to 21.90, 18.60 to 80.30, 14.93 to 30.49, and 33.53 to 109.72 µg g^−1^ FFW, respectively (Fig. 7). Red color genotype VA3 and VA13 had the highest total benzoic acids (58.40, 57.96 µg g^−1^ FFW) and total cinnamic acids (21.90, 21.27 µg g^−1^ FFW) while the green color genotype GRA1 showed the lowest total benzoic acids (14.64 µg g^−1^ FFW) and total cinnamic acids (3.96 µg g^−1^ FFW). Conversely, red color genotype VA3 had the highest total phenolic acids (80.30 µg g^−1^ FFW) followed by VA13 (79.23 µg g^−1^ FFW), while the green color genotype GRA1 showed the lowest total phenolic acids (16.60 µg g^−1^ FFW). In contrast, red color genotype VA13 had the highest total flavonoids (30.49 µg g^−1^ FFW) and total phenolic index (109.72 µg g^−1^ FFW) followed by VA3, while the green color genotype GRA1 showed the lowest total flavonoids (14.93 µg g^−1^ FFW) and total phenolic index (33.53 µg g^−1^ FFW) (Fig. 7). Total flavonoids, total phenolic acids and total phenolic index of our red color genotypes were higher than the content reported by Khanam *et al*.^37^ in *A*. *tricolor*. In plant tissues, the most extensively distributed phenolic acids are phenylalanine which finally synthesized as cinnamic acids^39^. In plants, although the most common forms of flavonoids are glycoside derivatives, occasionally it occurs as aglycone. Approximately, 60% of total dietary phenolic compounds are flavonoids^40,41^. In the plant kingdom, most predominant flavonoids are flavonols and naturally occurring most prevalent flavonols are glycosides of quercetin^40^. Klados and Tzortzakis^42^ noticed prominent variations in flavonoids and total phenolic acids content in *Cichorium spinosum*. Petropoulos *et al*.^43^ found significant phenolic acids and flavonoids differences among different *Cichorium spinosum*. Ahmed *et al*.^44^ observed significant variations in flavonoids and phenolic acids content of barley genotypes.

In the present study, we found remarkable phenolic acids such as salicylic acid, vanillic acid, protocatechuic acid, gallic acid, gentisic acid, β-resorcylic acid, *p*-hydroxybenzoic acid, syringic acid, ellagic acid, sinapic acids, chlorogenic acid, *trans*-cinnamic acid, *m*-coumaric acid, caffeic acid, *p*-coumaric acid, ferulic acid, and flavonoids such as rutin, hyperoside, isoquercetin, myricetin, quercetin, apigenin, kaempferol, and catechin in the red color amaranth genotypes, which was much higher compared to green color amaranth genotype. Our results corroborate with the results of Khanam and oba^32^ where they observed higher salicylic acid, vanillic acid, gallic acid, *p*-hydroxybenzoic acid, syringic acid, ellagic acid, chlorogenic acid, *m*-coumaric acid, caffeic acid, *p*-coumaric acid, ferulic acid, rutin and isoquercetin in the red amaranth genotype compared to green amaranth. Phenolic acids and flavonoids such as salicylic acid, vanillic acid, gallic acid, *p*-hydroxybenzoic acid, syringic acid, ellagic acid, chlorogenic acid, *m*-coumaric acid, caffeic acid, *p*-coumaric acid, ferulic acid, rutin, and isoquercetin obtained in this study were higher than the results of Khanam *et al*.^37^ in *A*. *tricolor*. The red color genotypes VA13 and VA3 had high pigments such as carotenoids, betalain, β-xanthin, and β-cyanin, and high antioxidant phytochemicals such as TFC, TPC, vitamin C, and TAC along with high phenolic acids such as salicylic acid, vanillic acid, protocatechuic acid, gallic acid, gentisic acid, β-resorcylic acid, *p*-hydroxybenzoic acid, syringic acid, ellagic acid, chlorogenic acid, sinapic acids, *trans*-cinnamic acid, *m*-coumaric acid, caffeic acid, *p*-coumaric acid, ferulic acid, and flavonoids such as hyperoside, rutin, isoquercetin, myricetin, quercetin, apigenin, kaempferol, and catechin. The genotypes VA13 and VA3 could be used as antioxidant and phenolic profile enriched high-yielding varieties for extracting the juice. The present investigation revealed that these two genotypes have abundant flavonoids, vitamins, phenolics, antioxidant leaf pigments, and antioxidants that offered new insight for detail pharmacological study.

### Correlation coefficient analysis

Correlation of antioxidant pigments and phytochemicals of red and green *Amaranthus* leafy vegetables are presented in Table 2. Highly significant positive associations of betalain, β-xanthin, and β-cyanin were exhibited among pigments and with TAC (ABTS^+^), TAC (DPPH), and TPC. Pigments of red and green amaranth (betalain, β-xanthin, and β-cyanin) showed strong antioxidant activity as all the pigments exhibited significant associations with TAC (ABTS^+^) and TAC (DPPH). Carotenoid pigments had significant correlation coefficients with TAC (ABTS^+^), TAC (DPPH), TFC, and vitamin C, while it showed insignificant associations with betalain, β-xanthin, and β-cyanin. Vitamin C exerted significant associations with TAC (ABTS^+^) and TAC (DPPH), whereas it exhibited negligible insignificant associations with betalamic pigments, TPC, and TFC. Our results corroborate with the results of Jimenez-Aguilar and Grusak^35^ for vitamin C in different amaranth species. The significant positive associations of carotenoids and vitamin C with TAC (ABTS^+^) and TAC (DPPH) also suggested a strong antioxidant activity. The significant associations of TPC and TFC were observed with TAC (ABTS^+^) and TAC (DPPH) indicating the strong antioxidant capacity of phenolics and flavonoids in red and green *Amaranthus* leafy vegetable. Alam *et al*.^45^ also reported corroborative results of TPC, carotenoids, TFC with TAC (FRAP) in salt-stressed purslane. Similarly, TAC (ABTS^+^) significantly associated with TAC (DPPH) that validated the measurement of antioxidant activity of two different methods in red and green *Amaranthus* leafy vegetables.

**Table 2:** Correlation coefficient for antioxidant leaf pigments, vitamin, TPC, TFC and TAC in three selected red and green *Amaranthus* leafy vegetable.

	β-xanthin (ng g^−1^)	Betalain (ng g^−1^)	Carotenoids (mg 100 g^−1^)	Vitamin C (mg 100 g^−1^)	TPC (GAE µg g^−1^ FW)	TFC (RE µg g^−1^ DW)	TAC (DPPH) (TEAC µg g^−1^ DW)	TAC (ABTS^+^) (TEAC µg g^−1^ DW)
β-cyanin (ng g^−1^)	0.95**	0.98**	0.34	0.16	0.85**	−0.67	0.85**	0.76*
β-xanthin (ng g^−1^)		0.79*	0.33	0.18	0.86**	−0.48	0.89**	0.75*
Betalain (ng g^−1^)			0.25	0.14	0.84**	−0.44	0.84**	0.83*
Carotenoids (mg 100 g^−1^)				0.94**	0.55	0.66*	0.82*	0.95**
Vitamin C (mg 100 g^−1^)					0.52	0.36	0.86**	0.96**
TPC (GAE µg g^−1^ FW)						0. 72*	0.96**	0.94**
TFC (RE µg g^−1^ DW)							0.89**	0.95**
TAC (DPPH) (TEAC µg g^−1^ DW)								0.98**

TPC, Total polyphenol content (GAE µg g^−1^ DW); TFC, Total flavonoid content (RE µg g^−1^ DW); TAC (DPPH), Total antioxidant capacity (DPPH) (TEAC µg g^−1^ DW); TAC (ABTS^+^), Total antioxidant capacity (ABTS^+^) (TEAC µg g^−1^ DW); *significant at 5% level, **significant at 1% level, (n = 3).

Red color *Amaranthus* genotypes are an excellent source of pigments, phytochemicals such as β- TPC, β-xanthin, TFC, cyanin, betalain, carotenoids, and vitamin compared to green color genotype. We also identified 24 phenolic acids and flavonoids such as vanillic acid, salicylic acid, protocatechuic acid, gallic acid, gentisic acid, β-resorcylic acid, *p*-hydroxybenzoic acid, syringic acid, ellagic acid, chlorogenic acid, sinapic acids, *trans*-cinnamic acid, *m*-coumaric acid, caffeic acid, *p*-coumaric acid, ferulic acid, hyperoside, rutin, isoquercetin, myricetin, quercetin, apigenin, kaempferol and catechin in the red color amaranth genotypes, which was much higher compared to green color amaranth genotype. Among them, we newly identified four flavonoids quercetin, catechin, myricetin, and apigenin in *Amaranthus* leaves. The red color genotypes VA13 and VA3 had abundant phenolic acids, pigments, flavonoids, antioxidant phytochemicals, and antioxidant could be used for extracting the juice. Correlation study revealed that all antioxidant constituents of red color amaranth had strong antioxidant activity. The present investigation revealed that two red color genotypes are an excellent source of antioxidants that offered huge prospects for detail pharmacological study. In the present study, the baseline data obtained from red and green amaranth could contribute to the scientists for the evaluation of pharmacologically active constituents.

## Methods

### Experiment materials, design, layout, and cultural practices

**Figure 8:**
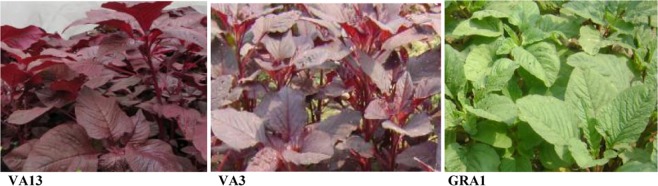
Three selected genotypes of red and green color *Amaranthus* leafy vegetable.

We selected three high yields and potentially antioxidant red color *A*. *tricolor* genotypes VA13 and VA3 as well as green color *A*. *lividus* genotype GRA1 (Fig. 8) from 120 genotypes^2–6,12–14^. Selected genotypes were grown in Bangabandhu Sheikh Mujibur Rahman Agricultural University in a randomized complete block design (RCBD) with four replications. The unit plot size of each genotype was 1 square meter. The spacing of each red and green color amaranth genotype was 20 cm between rows and 5 cm between plants. Recommended compost doses, fertilizer, and appropriate cultural practices were maintained. Thinning was done to maintain appropriate spacing between plants of a row. As a necessity, weeding and hoeing were done to remove the weeds. To maintain the normal growth of the crop proper irrigations were provided. At 30 days after sowing (DAS) of seed, leaves samples were collected. We measured all the parameters in four replicates.

### Chemicals

Solvent: acetone and methanol. Reagents: NaOH, dithiothreitol (DTT), caesium chloride, ascorbic acid, standard compounds of pure Trolox (6-hydroxy-2, 5, 7, 8-tetramethyl-chroman-2-carboxylic acid), standard compounds of pure phenolic acids, HPLC grade acetonitrile and acetic acid, gallic acid, rutin, folin-ciocalteu reagent, DPPH (2, 2-diphenyl1-picrylhydrazyl), ABTS^+^, aluminium chloride hexahydrate, sodium carbonate, potassium acetate, and potassium persulfate. All solvents and reagents were bought from Merck (Germany) and Kanto Chemical Co. Inc. (Tokyo, Japan).

### Estimation of leaf color

A color meter (TES-135A, Plus, Taiwan) was used to measure the color parameters C*, b*, L*, and a* in 15 replicates. The value of b* indicates yellow (+b*) or blue (-b*) color, a* indicates the degree of red (+a*) or green (−a*) color, L* indicates lightness, and. C* (Chroma) indicates the leaf color intensity. The chroma and L* values were calculated using the formula, Chroma C* = (a^2^ + b^2^)^1/2^ and Lightness, L* = 116 f (Y/Yn) − 16, respectively.

### β-Cyanin and β-xanthin content measurement

The leaves of red and green amaranth were extracted in 80% methyl alcohol having 50 mM ascorbate to measure β-cyanin and β-xanthin according to the method of Sarker and Oba^21^. A spectrophotometer (Hitachi, U-1800, Tokyo, Japan) was used to measure the absorbance at 540 nm for β-cyanin and 475 nm for β-xanthin, respectively. The results were expressed as the nanogram betanin equivalent per gram fresh weight (FW) for β-cyanin and nanograms indicaxanthin equivalent per gram FW for β-xanthin.

### Estimation of carotenoids

The leaves of red and green amaranth were extracted in 80% acetone to estimate carotenoids according to the method of Sarker and Oba^21^. A spectrophotometer (Hitachi, U-1800, Tokyo, Japan) was used to read the absorbance at 663 nm for chlorophyll *a*, 646 nm for chlorophyll *b*, and 470 nm for carotenoids, respectively. The data were calculated as mg carotenoids per 100 g FW.

### Estimation of vitamin C

The fresh red and green amaranth leaves were used to measure ascorbate (AsA) and dehydroascorbic acid (DHA) acid through a spectrophotometer. For pre-incubation of the sample and reduction of DHA into AsA Dithiothreitol (DTT) was used. AsA reduced Fe_3_^+^ to Fe_2_^+^ and estimation of AsA was made by the spectrophotometric (Hitachi, U-1800, Tokyo, Japan) measuring Fe_2_^+^ complexes with 2, 2-dipyridyl^21,46^. Finally, the absorbance of the sample solution was read. The data were recorded as mg vitamin C per 100 g of fresh weight (FW).

### Samples extraction for TPC, TFC, and TAC analysis

Thirty days after sowing (DAS) red and green amaranth leaves were harvested. For chemical analysis, the leaves were dried in the air in a shade. Forty ml of 90% aqueous methanol was used to extract 1 g of grounded dried leaves from each cultivar in a bottle (100 ml) capped tightly. A shaking water bath (Thomastant T-N22S, Thomas Kagaku Co. Ltd., Japan) was used to the extract for 1 h. The extract was filtered for determination of polyphenols, flavonoids, total antioxidant capacity.

### Estimation of phenolics

Method of Sarker and Oba^21,47^ was followed to estimate the total phenolic content of red and green amaranth using the folin-ciocalteu reagent with gallic acid as a standard phenolic compound. The folin-ciocalteu reagent was previously diluted 1:4, reagent: distilled water. In a test tube, 1 ml of diluted folin-ciocalteu was added to 50 µl extract solution and then mixed thoroughly for 3 min. One ml of Na_2_CO_3_ (10%) was added to the tube and stand for 1 h in the dark. A Hitachi U1800 spectrophotometer (Hitachi, Tokyo, Japan) was used to read the absorbance at 760 nm. A standard gallic acid graph was made to determine the concentration of phenolics in the extracts. The results are expressed as μg gallic acid equivalent (GAE) g^−1^ DW.

### Estimation of flavonoids

The AlCl_3_ colorimetric method^46,48^ was used to estimate the total flavonoid content of red and green amaranth extract. In a test tube, 1.5 ml of methanol was added to 0.1 ml of 10% aluminum chloride, 0.1 ml of 1 M potassium acetate, 2.8 ml of distilled water and 500 µl of leaf extract for 30 min at room temperature. A Hitachi U1800 spectrophotometer (Hitachi, Tokyo, Japan) was used to take the absorbance of the reaction mixture at 415 nm. TFC is expressed as μg rutin equivalent (RE) g^−1^ dry weight (DW) using rutin as the standard compound.

### Antioxidant capacity assay

Diphenyl-picrylhydrazyl (DPPH) radical degradation method^21,49^ was used to estimate the antioxidant activity. In a test tube, 1 ml of 250 µM DPPH solution was added to 10 µl of leaf extract solution (in triplicate) and 4 ml of distilled water and allowed to stand for 30 min in the dark. A Hitachi U1800 spectrophotometer (Hitachi, Tokyo, Japan) was used to read the absorbance at 517 nm. Method of Sarker and Oba^47,50^ was followed for ABTS^+^ assay. Exactly 7.4 mM ABTS^+^ solution and 2.6 mM potassium persulfate were used in the stock solutions. The two stock solutions were mixed in equal quantities and allowing them to react for 12 h at room temperature in the dark for preparation of the working solution. 2850 μl of ABTS^+^ solution (1 ml ABTS^+^ solution mixed with 60 ml methanol) was mixed with 150 μl sample of leaf extract and allowed to react for 2 h in the dark. Aa Hitachi U1800 spectrophotometer (Hitachi, Tokyo, Japan) was used to read the absorbance against methanol at 734 nm. The percent of inhibition of DPPH and ABTS^+^ relative to the control were used to determine antioxidant activity using the following equation:

$${\rm{Antioxidant}}\,{\rm{activity}}( \% )=({\rm{Abs}}.\,{\rm{blank}}-{\rm{Abs}}{\rm{.}}\,{\rm{sample}}/{\rm{Abs}}{\rm{.}}\,{\rm{blank}})\times 100$$

Where, Abs. blank is the absorbance of the control reaction [10 µl methanol for TAC (DPPH), 150 μl methanol for TAC (ABTS^+^) instead of leaf extract] and Abs. sample is the absorbance of the test compound. Trolox was used as the reference standard, and the results were expressed as μg Trolox equivalent g^−1^ DW.

### Samples extraction for HPLC and LC-MS analysis

10 ml of 80% methanol containing 1% acetic acid was added in 1 g of fresh leaves and homogenized thoroughly. 0.45 µm filter (MILLEX^®^-HV syringe filter, Millipore Corporation, Bedford, MA, USA) was used to filter the homogenized mixture. The mixture was centrifuged at 10,000 × g for 15 min. Flavonoids and phenolic acids were analyzed from the final filtrate.

### Flavonoids and phenolic acids analysis through HPLC

Sarker and Oba^27^ method was followed to determine flavonoids and phenolic acids using HPLC in red and green *Amaranthus* leaf samples. A variable Shimadzu SPD-10Avp UV–vis detector, LC-10Avp binary pumps, and a degasser (DGU-14A) were equipped with the HPLC system (Shimadzu SCL10Avp, Kyoto, Japan). A CTO-10AC (STR ODS-II, 150 × 4.6 mm I.D., Shinwa Chemical Industries, Ltd., Kyoto, Japan) column was used to separate phenolic acids and flavonoids. 6% (v/v) acetic acid in water (solvent A) and acetonitrile (solvent B) were pumped the binary mobile phase at a flow rate of 1 ml/min for a total run time of 70 min. a gradient program was used to run the system with 0–15% B for 45 min, 15–30% B for 15 min, 30–50% B for 5 min and 50–100% B for 5 min with the column temperature was maintained at 35 °C and the injection volume 10 μl. For simultaneous monitoring of benzoic acids, cinnamic acids and flavonoids, the detector was set at 370, 360, 254, and 280 nm. We compared retention time and UV–vis spectra with standards to identified the compound. The mass spectrometry assay method was used to confirm phenolic acids and flavonoids. HPLC quantified the sum total of all phenolic acids and flavonoids was denoted as the total phenolic index (TPI). The method described by Sarker and Oba^27^ was used to TPI from the HPLC data. All samples were prepared and analyzed in duplicate. The results were expressed as µg g^−1^ fresh weight (FW).

A JEOL AccuTOF (JMS-T100LP, JEOL Ltd., Tokyo, Japan) mass spectrometer fitted with an Agilent 1100 Series HPLC system and a UV–vis detector coupled on-line with an ElectroSpray Ionization (ESI) source to analyze the mass spectrometry with negative ion mode. The column elutes were recorded in the range of m/z 0–1000. Needle voltage was kept at −2000 V. The chromatographic conditions were optimized to obtain chromatograms with good resolution of adjacent peaks, for which a slight modification was made in the method reported by Sarker and Oba^27^. Extract constituents were identified by LC-MS-ESI analysis.

### Statistical analysis

Statistix 8 software was used to analyze the data for obtaining an analysis of variance (ANOVA). Duncan’s Multiple Range Test (DMRT) at 1% level of probability was used to compare the means. The results were reported as the mean ± SD of four separate replicates.

### Ethical statement

The lab and field experiments in this study were carried out as per guidelines and recommendations of “Biosafety Guidelines of Bangladesh” published by the Ministry of Environment and Forest, Government of the People’s Republic of Bangladesh (2005).

## Data Availability

Data used in this manuscript will be available to the public.
